# Assessment of a Potential UTI–Using Videos to Analyze a Simulated Nursing Home Experience

**DOI:** 10.1093/geroni/igaa057.1224

**Published:** 2020-12-16

**Authors:** Donna Owen, Alyce Ashcraft, Kyle Johnson, Huaxin Song

**Affiliations:** 1 Texas Tech University Health Sciences Center, Houston, Texas, United States; 2 Texas Tech University Health Sciences Cener, Lubbock, Texas, United States; 3 Texas Tech University Health Sciences Center, Lubbock, Texas, United States

## Abstract

In the past, urinary tract infections (UTIs) have been attributed to poor hygiene, the natural course of aging, an unfortunate corollary of nursing home (NH) residence, and a condition routinely treated empirically with antibiotics. Recent UTI management consensus statements foretell a very different future and support the need to consider all UTIs in the NH as complex infections. Improving assessment capabilities of the NH nurse workforce is essential for improving quality of care. This study aimed to determine how, using simulation, licensed vocational nurses (LVNs) integrated a mobile decision-support app (MDS-app) into assessment of a NH resident with a potential UTI. The MDS-app directed the LVN to examine or question the resident (mannequin) to identify signs and symptoms developed as part of a simulated clinical scenario. MDS-app items were based on UTI practice guidelines. A descriptive, participant observation design (video-taped) was used with ten practicing LVNs. An observation checklist was used to examine audiovisual recordings and included frequency of verbal interaction (17.9+/-7.2), and eye contact (10.6+/-4.1). Participants (47%) were “glued to” the MDS-app without making resident eye contact or touching residents during the assessment. 60% of participants deviated from the app to ascertain urine odor and color; irrelevant symptoms for UTI diagnosis. Assessments required 11.20 (+/-4.67) minutes to complete. The MDS-app provided LVNs with needed focus on data driven by guidelines and not individual LVN preferences. Training LVNs should focus on integration of communication, assessment skills, and MDS-app use for evidence-based data collection as a basis for UTI treatment decisions.

